# On the Internal Use of Plumbi Acetas

**Published:** 1853-10

**Authors:** W. Johnson

**Affiliations:** White House, Hunterdon County, N. J.


*On the Internal use of Plumbi Acetas.* By W. Johnson, M D.,

The discrepancies of medical testimony, with respect to the value of therapeutic agents, are frequently so great as to extort from us the humiliating confession "ars nostra conjecturalis est." This truth is strikingly demonstrated in the article now under consideration. By one set of practitioners it meets with unqualified reprobation as an irritant poison; by another portion of the profession it is highly lauded as a perfectly safe remedy, and one possessed of most invaluable powers. How are we to reconcile such contrarity of views? Only by making allowance for lack of faithful observation, patient investigation and matured conclusion. Truth often lies between extremes.

It may be useful to examine the merits of this controversy, and hear the allegations of both sides. The limits of an essay will not, however, permit me to go into an elaborate examination of the mass of testimony on this subject, and perhaps we had better let a few on each side, be the exponents of the views of their respective parties.

Hear first the negative side, in the person of Dr. Meigs, the learned professor of Jefferson Medical College. "Sugar of lead is thought to be a very powerful article in the treatment of these hemorrhages. I do not like it, nor do I believe much in it; perhaps I have not dared to give it in excessive quantities, and having very rarely exhibited more than three grains in combination with opium, for the dose, I am not sensible that I have ever been much struck with its hæmostatic powers, though I have made use of it many times, yet less frequently of late than in former years."

The late Professor Hosack, of New York, if I am not greatly in error, reprobated, altogether, the internal use of this article.

Here is strong and weighty negative testimony, and dogmas emanating from such sources must have had a controlling influence on American practice, and lead has not consequently been administered in the United States with so bold a hand as by our transatlantic brethren. The names of Hosack and Meigs are in themselves a host; but "magna est veritas et prævalebit."

Therefore, let us hear what is said on the opposite side of this question. First, with respect to the safety of the article. Dr.

Henderson, of Edinburgh, gave in bronchitis, to restrain inordinate secretion from the bronchia, four scruples of the lead in ten days, to a child of six years of age, without any unpleasant effect whatever. The blue line on the margin of the gums was noticed, but no constipation of the bowels took place.

Surgeon Kirby, of Dublin, prescribed one grain of acetas plumbi every two hours, in a case of diabetes melitus, and with very good effect The medicine was taken for one month.

Surgeon Sweeting states "that a woman was admitted into Guy's Hospital, having swallowed one and a half ounces of sugar of lead, dissolved in water, from which she recovered and left the hospital in five days."

"Mr. Gorringe reported two cases in which two girls swallowed by mistake, one ounce of acetate of lead, and another in which a girl swallowed one drachm, and all recovered."

"Dr. Hinding gives a case in which a girl swallowed three drachms; she too recovered."

"Dr. Iliff records four cases in which individuals swallowed each an ounce of the acetate of lead; and Dr. Evans one, in which three drachms were taken, and all in a very short time recovered."

All these cases go to show in a very remarkable manner, that the danger from the internal use of lead has been overrated.

Surgeon Sweeting records a case in his own practice, in which he administered five grains of the acetate, by enema, to a child of seven years of age, laboring under typhus, in which profuse hemorrhage from the bowels was promptly arrested, and the child fully recovered.

In uterine hemorrhage he gives the article with a bold hand. In one instance, five grains were repeated every hour for forty-eight hours, and it was then given every four hours, and continued for a fortnight with good effect. In this case the patient took in sixteen consecutive days 576 grains of the lead.

Nan Swieten, he tells us, administered sixty grains daily, for ten days. The largest single dose administered by the reporter, was twenty grains. He states that Mr. Daniel gave ten grains three times a day, from which he saw no other ill effect than a slight colic.

"The boldness," says our reporter, "with which I have administered the acetate, is not the result of mere speculation, nor of observations made in one month, or one year, but rather of patient and careful watching for the long period of *fourteen* years, during which time I have never met with an instance, in which the ill effect usually ascribed to lead have followed the administration of it." He advises the article to be given in simple distilled water.

Dr. Reginal Burridge, senior physician of the Somerset and Taunton Hospital, gives an interesting case of a young lady who attended upon him in an epidemic dysentery. She was attacked with profuse hemorrhage from her bowels. "When arrived at the house he was shown three large evacuations; the first two contained a pint each, of loose coagula, the third was a solid coagulum, fourteen inches long, and formed a complete mould of the colon. She was cold and pulseless, the eyes fixed and glazed, the tongue cold, and the cold damps of death had already descended upon her. The anæmic state was very marked, including the rare and sighing, almost sobbing, respiration."

He gave her a couple of teaspoonfuls of brandy, with a few drops of hartshorn, and very soon a scruple of the acetate of lead. She soon passed another coagulum, and he immediately gave her a small starch enema containing half a drachm of lead. In a short time he gave the second scruple of the acetate, and in one hour after the third scruple. She convalesced without further hemorrhage, and without any unpleasant symptom, and completely recovered.

Dr. Burridge relates another interesting case. It was that of an old soldier of eighty years of age, who passed daily large sphacelated portions of the colon and rectum, the mutilated intestine protruding through the anus in large gangrenous shreds like the fingers of a glove. Eight grains of the acetate every three hours, with the local use of the article, acted like a charm, and restored the old man to as good health as is ever enjoyed by a person of this age.

Dr. Burridge states, that during twenty-one years of ample experience with the article, he has never witnessed a single untoward symptom from its therapeutic use

The doctor always gives the acetate in a fluid form; *never* without acetic acid, and very rarely without Battley's liq. opii sedativ. His large hospital practice has enabled him to determine that the efficacy of the article is increased by combining it with opium.

The last two authorities which I have quoted—namely, Surgeon Sweeting and Dr. Burridge, bear most satisfactory testimony to the great remedial powers of the acetas plumbi, and their united experience, extending over thirty-five years, goes far to establish the safety of the therapeutic use of the article. They both declare that they never in their hands, witnessed one single unpleasant effect from the use of the article. It should be borne in mind too, that they gave maximum doses of the acetate, and continued its use much longer than would generally be thought either safe or prudent.

My own experience will somewhat qualify that of these two eminent men, particularly with respect to the safety of the article.

I have for more than forty years prescribed the acetas plumbi, and with undiminished confidence in its remedial powers. I wish, however, that I could add that I have never witnessed any unpleasant consequences from its administration. Four times have most painful solicitudes been awakened by the production of saturine disease, from the internal use of this article ; twice in the form of colica pictonum, and twice in that of paralysis. These results have rendered me more cautious in the use of the article, but have not diminished my faith in its remedial powers. The first of these adverse cases occurred about thirty-five years since. The patient had had two or three attacks of hæmoptysis for which I prescribed the acetas plumbi, and with most decided benefit. She resided about five miles from me, and after her recovery from her last attack, I left a quantity of the medicine with her, that she might use it if a return of the disease should render it necessary before I could visit her. She experienced some promonition of a return of the hæmoptysis, and thinking that the article might possess preventive, as well as curative powers, she commenced taking it, and continued so long with it as to produce a most violent attack of colic. For several days her sufferings were very great. Her disease yielded to sulph. magnes. and opium, and the irritation had been so completely transferred from the lungs to the intestinal canal, that she had no return of hæmoptysis, nor has she had to this day.

The next case of ill effects from the lead occurred in a female patient near the climacteric period. It was prescribed for menorrhagia. The lead was used for some time. The attack of colic was very severe, but yielded to the same treatment pursued in the former case, and with complete success, though not until after the lapse of several days.

The third case was menorrhagia. The acetas plumbi was used about a fortnight. The bowels became confined, but pains, something like neuralgia, seized upon the loins, back part of the pelvis, and upper part of the thighs. They were most excruciating, and were attended with considerable loss of motor power in the muscles, which were the seat of pain. The case was treated as the former, with the addition of frequent cupping and anodyne liniments. The case was not very protracted, and the recovery was complete.

The fourth and last case was also menorrhagia. The regular turns of the catamenia were very protracted, and the discharge was so profuse as to blanch the patient. The lead was prescribed, and used for more than a fortnight. Severe pain, resembling neuralgia, was experienced in the loins and down the thigh, following the course of a sciatic nerve, together with great loss of muscular power. One side was more affected than the other. The characteristic blue line at the margin of the gums, indicative of constitutional impregnation with lead, was present is this case in a very remarkable degree. The case was treated as the last, and with complete success, and was not very tedious. The condition of the catamenia vastly improved.

I have never used the lead with so bold a hand as have the practitioners of Great Britain. I never, but once, gave a drachm in twenty-four hours. My usual manner of prescribing the article, is to give three grains every twenty minutes or half hour in urgent cases, say post partum hemorrhage—hæmoptysis, &c., until an effect be produced. In ordinary cases, I give it every two, three or four hours, generally combined with opium, but of late I have used the article alone. I have prescribed the acetas plumbi in diarrhœa, dysentery, and cholera infantum with very pleasing results; but it is in the hemorrhages that I have most frequently employed the article. In hæmoptysis there is no article to which I am more partial. In epitaxis, too, I have seen most excellent effects from the employment of the lead. But it is particularly in uterine hemorrhage that I have witnessed the powers of the acetas plumbi. In a practice of more than forty years, I have never lost a patient with uterine hemorrhage; either simple menorrhagia, post partum, or unavoidable hemorrhage. I state not this in the spirit of self-commendation; but cannot forbear recording it as a grateful remembrance of the good hand of my God upon me.

In the treatment of all these cases, I have trusted more to the acetas plumbi, than to any other therapeutic agent. But the tampon, the introduction of the hand in utero, cold applications, &c., have been used in their appropriate places. In post partum hemorrhage, I often give the ergot in preference to the acetate of lead, but I also frequently administer them conjointly. The indication in these cases evidently is, to diminish the calibres of the uterine vessels, and to render them more turtuous, and thus throw impediments to the exodus of the blood; this alone can be effected by the active contraction of the womb, and ergot is well adapted to the fulfilment of this indication. I also give the preference to argot in ante-partum hemorrhage, *when the period of gestation and the state of the os uteri indicate such preference.* But in very many of both these sets of cases, well directed manipulations with the hand in utero is the measure best calculated to insure the safety of the patient.

I have sometimes too, in simple menorrhagia given the preference to *monecia*, which by the by I consider a most invaluable article. I have also used the tannin with most pleasing effects. These articles should be given in as large a dose as the stomach will well bear. The ol. terebith. and sulph. alum. I have also used with decided good effect. I have used and still do use the foregoing articles, but my partialities are towards the lead. In some cases of hemorrhage we have no good substitute for the lead. I allude particularly to those fearful discharges of blood, which sometimes occur in enteric fever. Lead is here a host, and should be given with the most liberal hand, both per orem et anum. The ol. terebinth. is also a useful adjunct in these cases.

But I have extended my remarks farther than I had intended at the commencement.. Indulge a few inferences, and I have done.

1st. Lead is a valuable therapeutic agent.

2d. The danger from the use of it has been overrated.

3d. It is not inoxious.

4th. Therefore the patient to whom it is given should be carefully watched, and as soon as the blue line of Burton makes its appearance, let the article be discontinued.

5th. There appears to be no more danger from large doses of the acetas plumbi than from smaller ones—contrast my experience of the article with that of Sweeting and Burridge.

*White House, Hunterdon County, N. J.*

